# When Nature Meets Oncology: Unraveling Herb–Drug Interactions in Cancer Therapy

**DOI:** 10.3390/ijms262110494

**Published:** 2025-10-29

**Authors:** Ayush Gandhi, Samip Master, Viraj Bhise

**Affiliations:** 1 Department of Hospital Medicine, The University of Texas MD Anderson Cancer Center, Houston, TX 77030, USA; vrbhise@mdanderson.org; 2 Texas Oncology, Webster, TX 77598, USA; samip.master@usoncology.com

**Keywords:** natural products, herb-drug interactions, cancer, chemotherapy, targeted therapy, immunotherapy, cytochrome P450

## Abstract

Natural product (NP) use by patients alongside their conventional cancer therapies is ubiquitous. This common, yet often hidden, practice can potentially contribute to significant patient harm, given the narrow therapeutic window of most anticancer drugs. This review takes on this challenge directly, moving past theoretical concerns to summarize current clinical evidence on interactions between widely used NPs and modern cancer treatments, including chemotherapy, targeted therapy, and immunotherapy. We break down the key pharmacokinetic (PK) mechanisms, such as the disruption of cytochrome P450 enzymes, and the pharmacodynamic (PD) effects that can either help or hinder treatment. By examining both well-established clinical interactions and those supported by preliminary or preclinical findings, we highlight how NPs may alter the effectiveness of anticancer medications and where evidence remains uncertain. Lack of reliable safety information for NPs along with widespread use of these products by patient populations has the potential to impact clinical care and patient outcomes significantly, frequently causing harm. We advocate for improved patient-provider communication and additional evidence-based research to address this gap in literature. The majority of reported interactions are based on preclinical or limited clinical evidence. A more rigorous evidence base including real-world data and clinical trials is urgently needed to guide practice.

## 1. Introduction

### 1.1. Clinical Consideration in Concurrent Use

The use of natural products (NPs), from herbal medicines to dietary supplements, is a global phenomenon among patients with cancer. Worldwide, an estimated 22% of oncology patients use herbal medicines, with rates varying from 17–21% in North America and Europe to as high as 40% in Africa [1]. Some studies report even higher use, with nearly half of patients (46%) in certain cohorts regularly incorporating herbal products alongside their conventional cancer treatments [2]. The reasons are deeply personal: patients hope to improve their quality of life, bolster their immune system, manage symptoms, and ease the harsh side effects of treatment [3].

### 1.2. Perceived Safety of Natural Products and the Risk of Non-Disclosure

A pervasive and potentially dangerous myth fuels this trend: the belief that “natural” automatically means “safe”. This assumption leads to a critical communication breakdown, as more than half of all cancer patients do not tell their doctors or pharmacists about their NP use [3]. They may feel it is irrelevant to their care, or they may fear that their providers will be dismissive or simply lack the knowledge to offer guidance [4]. The result is that clinicians are often flying blind, completely unaware of potent bioactive compounds being taken with prescribed therapies, introducing a hidden and unpredictable variable into patient care non-disclosed supplements that can potentially impact clinical care

### 1.3. A High Stakes Environment

Non-disclosed natural products can be especially dangerous in oncology. Most cancer drugs, especially cytotoxic chemotherapies, operate within a thin therapeutic window where the line between an effective dose and a toxic one is incredibly fine. In practice, even small shifts in drug exposure can cause a treatment to fail or trigger life-threatening side effects that may limit its usefulness in the future. When a patient’s undisclosed supplement use collides with this delicate pharmacology, it creates a significant patient safety risk. What happens next? An adverse event or poor response might be blamed on the drug or the disease, leading to misguided decisions like dose reductions, treatment delays, or even stopping a lifesaving therapy all while the true culprit, an herb–drug interaction, remains invisible.

### 1.4. From Uncertainty to Evidence-Based Guidance

For too long, the risks of these interactions have been dismissed as merely “theoretical,” with systematic reviews noting a lack of direct clinical proof [5]. This scarcity of hard data creates a cycle of uncertainty: clinicians hesitate to give firm advice, and patients turn to unreliable sources, further widening the communication gap. Consequently, the true incidence of harm is almost certainly underrecognized and underestimated. This review presents a summary of current evidence on interactions between common NPs and today’s cancer therapies such as chemotherapy, targeted drugs, and immunotherapy. We also explore underlying mechanisms and highlight examples.

While several recent reviews have mapped out the mechanistic landscape of herb–drug interactions in cancer therapy [4,6], what we have tried to do here is a little different. Rather than stopping at the biological underpinnings, our emphasis is on how this knowledge can actually guide real-world decisions at the bedside. In contrast to prior studies [3,6], we have built the discussion around a structured risk-stratification framework that weighs the strength of the evidence. We then connect these mechanisms to treatment contexts where clinicians most often face uncertainty, such as patients receiving TKIs or ICIs. The goal is not just to describe interactions but to turn them into something actionable and a practical reference point that supports decision-making in everyday oncology practice.

### 1.5. Dietary Versus Pharmacologic Exposure

A critical distinction must be made between naturally occurring dietary intake of NPs (e.g., turmeric in food) and high-dose supplemental forms. The metabolic and pharmacologic behaviors differ substantially such as bioavailability, excipients, and concentration all modulate interaction risk. Throughout this review, ‘supplemental use’ refers to concentrated formulations exceeding typical dietary exposure. Recognizing this distinction prevents unnecessary alarm and guides nuanced clinical counseling.

## 2. Materials and Methods

A structured literature search was performed following PRISMA-ScR guidelines. Databases searched included PubMed, Scopus, and Web of Science from January 2000 to June 2025 using the following key terms: (“natural products” OR “herbal medicines” OR “dietary supplements”) AND (“cancer” OR “chemotherapy” OR “tyrosine kinase inhibitors” OR “immunotherapy”) AND (“drug interaction” OR “pharmacokinetic” OR “pharmacodynamic”).

Inclusion criteria comprised original human studies (clinical trials, observational studies, pharmacokinetic analyses), systematic reviews, and preclinical mechanistic reports with translational relevance. Exclusion criteria were non-oncology studies, case reports without pharmacologic plausibility, and non-English publications.

Two authors independently screened titles and abstracts, followed by full-text review; disagreements were resolved by consensus. Data extraction captured study design, cancer type, natural product, concomitant therapy, outcome, and level of evidence.

Study quality was assessed using the Newcastle–Ottawa Scale (observational studies) or Cochrane Risk-of-Bias tool (randomized trials).

## 3. Mechanism of Drug Interactions

Interactions between NPs and anticancer drugs frequently occur through predictable biological pathways. They fall into two major categories: pharmacokinetic (PK) interactions, which change how much of a drug is in the body, and pharmacodynamic (PD) interactions, which alter the drug’s effects.

### 3.1. Pharmacokinetic (PK) Interactions: Altering Drug Exposure

PK interactions influence the absorption, distribution, metabolism, and excretion (ADME) of drugs, thereby altering their concentration in the body and at the site of action.

#### 3.1.1. Altering Drug Metabolism

The most common and critical PK interactions involve the body’s primary detoxification system: the cytochrome P450 (CYP) family of enzymes. These enzymes, located mostly in the liver and gut, are responsible for breaking down over half of all chemotherapy agents and the majority of oral targeted drugs [1]. NPs can interfere with them in two main ways.

Enzyme induction: Some NPs effectively accelerate drug metabolism. St. John’s wort (*Hypericum perforatum*) is the poster child for this effect. Its active compound, hyperforin, activates a receptor that ramps up the production of the CYP3A4 enzyme, causing drugs to be cleared from the body much faster. This can drop drug levels so low that the treatment becomes ineffective [7,8,9].

Enzyme inhibition: Other natural products inhibit drug-metabolizing enzymes, particularly CYP3A4, leading to reduced metabolic clearance and increased systemic drug exposure. Grapefruit juice, for instance, contains compounds that shut down CYP3A4 in the gut, which can cause oral drug levels to skyrocket to toxic concentrations. Other well-known inhibitors include berberine (found in goldenseal) and curcumin [1].

These interactions are well demonstrated for some compounds, such as St. John’s wort and grapefruit juice, and are suggested by preclinical or small PK studies for others. This variability demonstrates the importance of distinguishing interactions with strong clinical support from those that remain largely theoretical.

#### 3.1.2. Rerouting Drug Transport

Beyond metabolism, transport proteins act as cellular gatekeepers, controlling where drugs can and cannot go. Efflux pumps like P-glycoprotein (P-gp) actively push drugs out of cells in the gut, liver, and brain. St. John’s wort induces these pumps, further accelerating drug clearance, while other NPs can inhibit them, leading to higher drug exposure and potential toxicity [1].

#### 3.1.3. Effects on Phase II Metabolism

After initial processing (Phase I), drugs are tagged for removal by Phase II enzymes like UGTs. These enzymes are crucial for detoxifying drugs like irinotecan. NPs that interfere with this final step can also have a major impact on a drug’s safety and efficacy [1].

### 3.2. Pharmacodynamic (PD) Interactions: Changing Drug’s Effects

PD interactions occur when an NP alters a drug’s effect at the molecular level, without changing its concentration. Clinicians often note that the challenge lies in distinguishing the rare but clinically significant effects from theoretical or preclinical findings

Synergy: Some NPs can actually help anticancer drugs work better. For example, preclinical studies suggest that ginseng may suppress pro-survival signaling pathways in cancer cells, potentially increasing their susceptibility to agents like 5-FU and cisplatin. However, clinical confirmation of this effect remains limited [10].

Antagonism: Conversely, some NPs may directly sabotage a drug’s mechanism. A long-standing concern is that high-dose antioxidant supplements could neutralize the reactive oxygen species (ROS) that many chemotherapies and radiation need to kill cancer cells. Mechanistic studies demonstrate that the green tea polyphenol EGCG binds directly to bortezomib, potentially neutralizing its activity. Although the biochemical interaction is well characterized, clinical evidence is limited [11].

Given these mechanistically plausible but clinically unproven concerns, the routine use of high-dose antioxidant supplements during chemotherapy or radiation should be approached with caution and is generally discouraged outside of a research setting, particularly in treatment contexts that rely on oxidative stress for therapeutic efficacy.

Toxicity protection: Not all interactions are bad. Some NPs can selectively protect healthy tissues from treatment side effects. For instance, the Japanese Kampo medicine *Hangeshashinto* has been shown in clinical studies to reduce chemotherapy-induced diarrhea and mouth sores. Similarly, ginseng may help shield the heart and kidneys from chemotherapy damage [10].

Understanding the difference between PK and PD interactions is critical. A PK interaction that changes drug levels could theoretically be managed with a dose adjustment. But a direct PD antagonism, like EGCG neutralizing bortezomib, is an absolute conflict that cannot be fixed by changing the dose, making it one of the most dangerous interactions in oncology.

**Figure 1:**
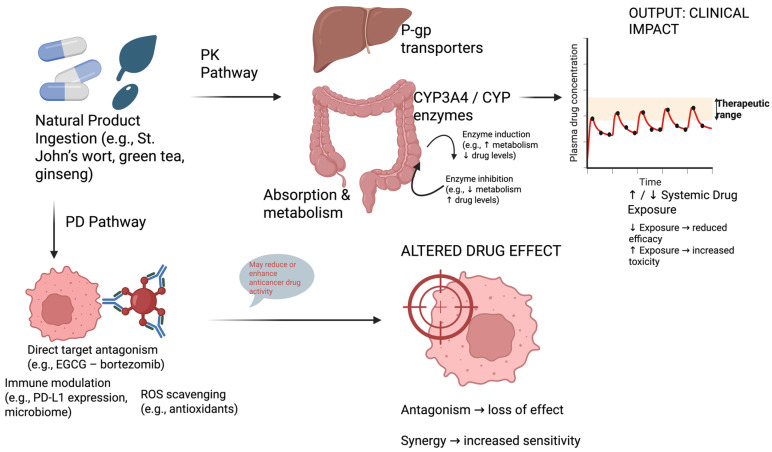
Schematic illustration of pharmacokinetic (PK) and pharmacodynamic (PD) interaction pathways between natural products and anticancer drugs. PK interactions typically involve modulation of absorption, metabolism, and transport (e.g., CYP3A4, P-gp), altering systemic drug exposure. PD interactions may involve direct target antagonism, immune modulation, or reactive oxygen species scavenging, affecting therapeutic efficacy or toxicity.

A schematic representation of PK and PD interaction pathways is provided in Figure 1 to illustrate how natural products can alter drug exposure and therapeutic effects. Table 1 demonstrates key pharmacodynamic interactions of natural products in oncology.

## 4. The Therapeutic Potential of Natural Products in Oncology

While it is crucial to focus on risk, it is equally important to recognize that NPs can also offer real therapeutic benefits. A growing body of evidence shows they can help patients manage side effects, potentially enhance conventional therapies, and in some cases, fight cancer directly [6]. This dual potential for benefit and harm highlights the importance of conducting systematic, evidence-based research on these supplements.

### 4.1. Easing the Burden of Treatment

One of the most compelling roles for NPs in oncology is in supportive care helping patients cope with the debilitating side effects of treatment.

Ginger (*Zingiber officinale*): A trusted remedy for nausea, ginger has been validated in multiple clinical trials for reducing chemotherapy-induced nausea and vomiting (CINV), with daily doses of 0.5–1.0 g being most effective [15].

Ginseng (*Panax ginseng* and *Panax quinquefolius*): Cancer-related fatigue can be relentless. A major Phase III trial found that American ginseng (2000 mg daily) significantly reduced fatigue in patients undergoing active therapy [16]. Preclinical and early clinical data also suggest ginseng may protect against chemotherapy induced cardiotoxicity [17].

Astragalus (*Astragalus membranaceus*): When combined with chemotherapy for non-small cell lung cancer, astragalus injections have been linked to better quality of life and relief from fatigue and nausea [4].

Medicinal mushrooms: Species like Maitake (*Grifola frondosa*) are often used to lessen chemotherapy side effects and improve appetite [18].

### 4.2. Working in Synergy with Conventional Drugs

Beyond symptom relief, some NPs may act as allies to conventional drugs, boosting their anticancer effects [19]. This can happen through complementary mechanisms for example, targeting signaling pathways distinct from those affected by standard therapies [20]. Preclinical studies show that ginseng can increase the cytotoxicity of 5-FU and cisplatin, and may even help overcome multidrug resistance [10]. Similarly, curcumin has been shown to sensitize tumor cells to chemotherapy while protecting normal tissues [14].

While the potential for adverse herb–drug interactions must be taken seriously, it is equally important to recognize that several natural products have demonstrated meaningful therapeutic benefit in supportive oncology. Clinical trials provide encouraging data for agents such as ginger for chemotherapy-induced nausea and vomiting (CINV), ginseng for cancer-related fatigue, and astragalus for symptom relief and quality of life. Presenting these benefits alongside their potential PK and PD risks allows for more nuanced, evidence-based discussions with patients. Table 2 summarizes selected natural products with both documented therapeutic benefits and interaction considerations.

Integrating these supportive roles within the same clinical framework that governs risk assessment enables balanced decision-making. The intent is not to deter use of beneficial NPs such as ginger or ginseng but to embed their consideration within the same structured dialogue used for potential risk management.

### 4.3. Legacy of Natural Product Drug Discovery

We cannot forget that natural products are the original source of many of our most important cancer drugs. Paclitaxel (from the Pacific yew tree) and the vinca alkaloids (from the rosy periwinkle) are cornerstone chemotherapies derived directly from nature [4,20]. Current research continues to explore new NPs with direct anticancer potential:

Curcumin: Extensive preclinical studies suggest curcumin disrupts multiple signaling pathways linked to cancer progression, including proliferation, angiogenesis, and metastasis [21].

Medicinal mushrooms: Compounds from mushrooms such as Turkey Tail (*Trametes versicolor*) and Maitake (*Grifola frondosa*) have demonstrated immunomodulatory and direct antitumor activity PSK, a polysaccharide extract of Turkey Tail, is already an approved anticancer adjuvant in Japan, where it has been shown to improve survival when added to standard therapies [18].

## 5. Interactions with Conventional Chemotherapy

Cytotoxic chemotherapy is still a mainstay of cancer treatment, but its narrow therapeutic window makes it highly susceptible to natural product drug interaction through both pharmacokinetic (PK) and pharmacodynamic (PD). The evidence, however, is a patchwork of rigorous trials, conflicting lab studies, and isolated case reports, making it difficult for clinicians to offer clear advice.

### 5.1. St. John’s Wort (Hypericum perforatum)

St. John’s wort is the most well-documented and clinically significant herb-drug interaction in oncology. By strongly inducing both the CYP3A4 enzyme and the P-gp transporter, it drastically lowers the exposure of many anticancer drugs. A landmark trial showed that St. John’s wort slashed exposure to the active metabolite of irinotecan by 42%, a drop that directly correlated with a loss of the drug’s therapeutic effect [22]. Similar findings have been reported with docetaxel, where St. John’s wort increased clearance and reduced plasma levels [23].

Recent pharmacovigilance and real-world pharmacoepidemiologic analyses continue to affirm this risk, with large-scale datasets identifying St. John’s wort as a consistent signal for CYP3A4-mediated interactions across oncology cohorts. These findings corroborate early clinical evidence while demonstrating the persistence of this risk in contemporary cancer pharmacotherapy [24]. The evidence is so strong that the concurrent use of St. John’s wort with chemotherapy is strictly contraindicated.

### 5.2. Curcumin (Curcuma longa)

Curcumin, the active compound in turmeric, has a much more complicated and contradictory profile. Some lab studies raise concerns that it could interfere with chemotherapy prodrugs or neutralize the ROS needed for certain drugs to work [25]. Yet other studies suggest it can make cancer cells more sensitive to chemotherapy while protecting healthy tissues from side effects, like 5-FU induced gut toxicity [1]. Its metabolic effects are just as confusing, with reports of both inhibiting and inducing various CYP enzymes. To our knowledge, no large, randomized trial has provided definitive answers, leaving clinicians reliant on case reports and small studies

Taken together, the conflicting mechanistic data and absence of adequately powered clinical trials make it difficult to offer definitive guidance; however, in practice, high-dose curcumin or antioxidant supplementation during active cancer therapy is best avoided unless supported by clear evidence or administered under clinical supervision.

### 5.3. Ginseng (Panax ginseng)

Ginseng has long been studied as both a supportive therapy and a potential risk. Lab research shows it can boost the killing power of chemotherapy, overcome drug resistance, and reduce toxicities like cardio and nephrotoxicities [10]. However, ginseng also inhibits several CYP enzymes, and case reports are concerning. In one case, a ginseng-containing energy drink was linked to severe liver toxicity in a patient who had tolerated the cancer drug imatinib for years [26]. This highlights the unpredictable danger of interactions, especially from multi-ingredient commercial products.

### 5.4. Green Tea (Camellia sinensis) and Garlic (Allium sativum)

Green Tea: The main risk here is direct antagonism. Its major polyphenol, EGCG, binds to and inactivates the proteasome inhibitor bortezomib. Green tea may also inhibit enzymes that process drugs like irinotecan [27].

Garlic: The primary concern with garlic supplements is an increased risk of bleeding. Its antiplatelet properties can worsen chemotherapy-induced low platelet counts or interact dangerously with anticoagulants [28,29].

### 5.5. Other Notable Interactions

Other common herbs also demand caution. Echinacea has been linked to severe thrombocytopenia in a patient on etoposide [30]. While black cohosh may protect cancer cells from cisplatin [31]. Milk Thistle has shown mixed effects on drug metabolism and may increase tamoxifen levels [32].

Patients often use complex multi-herb formulas from traditional medicine or proprietary blends whose ingredients are a mystery. The pharmacological consequences of these combinations are almost entirely unstudied, representing a vast and uncharted territory of potential risk.

## 6. Interactions with Targeted Therapies

Oral targeted therapies, especially tyrosine kinase inhibitors (TKIs), have revolutionized cancer care. But the very things that make them convenient, their oral administration and reliance on specific pharmacokinetic (PK) pathways also make them exceptionally vulnerable to natural products (NP) interactions.

### 6.1. Tyrosine Kinase Inhibitors (TKIs): A High-Risk Class

Most TKIs, such as imatinib and erlotinib, are processed by the same duo: the CYP3A4 enzyme and the P-gp efflux pump [33,34]. Unlike IV chemotherapy, which faces its first metabolic challenge in the liver, oral TKIs are first exposed to these systems in the gut wall. This means that even NPs that are poorly absorbed into the bloodstream can have a profound effect on drug levels right at the site of absorption

CYP3A4 induction: The danger of combining TKIs with a strong inducer like St. John’s wort is well-established. Clinical data show it increases the clearance of imatinib by 43%, causing a 30% drop in drug exposure—a reduction that could easily lead to treatment failure and drug resistance [35].

CYP3A4 inhibition: On the flip side, inhibitors can cause drug levels to climb to dangerous heights. Grapefruit juice, for instance, markedly increases the bioavailability of nilotinib and sirolimus, raising the risk of toxicities such as myelosuppression or QTc prolongation [36]. Other potent inhibitors including goldenseal (berberine) and Kava Kava pose similar risks and should be strictly avoided by patients receiving TKIs [37,38].

### 6.2. The Paradox of Natural Kinase Inhibitors

To add another layer of complexity, many NPs naturally contain compounds that inhibit the very same kinase pathways targeted by modern drugs. Flavonoids like quercetin and stilbenoids like resveratrol are actively being researched for their own anticancer properties. This creates a paradox: a compound that shows promise in a lab study could, when taken as an over-the-counter supplement, directly interfere with a life-saving prescription TKI. This disconnect between research and clinical safety can be deeply confusing for patients, making clear communication all the more critical [39].

## 7. Interactions with Cancer Immunotherapies

Immune checkpoint inhibitors (ICIs) are one of the newest pillars of cancer care, working not by killing cancer cells directly but by unleashing the patient’s own immune system. Because of this unique mechanism, NP interactions in this space are fundamentally different, they act on the immune system itself and the tumor microenvironment (TME). Potential pharmacodynamic (PD) modulation is of particular relevance here, as these agents act on immune signaling rather than on classical pharmacokinetic (PK) pathways.

### 7.1. Modulating the PD-1/PD-L1 Axis

The success of ICIs hinges on the interaction between T-cells and tumor cells at the PD-1/PD-L1 checkpoint. Emerging lab data suggest that natural phytochemicals can influence this critical axis.

Potential synergy: Preclinical studies indicate that compounds like curcumin, resveratrol, and quercetin have been shown to reduce the expression of PD-L1 on tumor cells [40]. In theory, this could release the “brakes” on the immune system and help ICIs work better, potentially turning immunologically “cold” tumors into “hot” ones that are more responsive to treatment however clinical trials needed to validate these effects.

Potential antagonism: However, the effects are not always positive. Other agents, and even resveratrol in some studies, have been shown to increase PD-L1 expression, which could counteract the benefit of ICIs [40].

### 7.2. Current Status

While the clinical evidence base remains limited, several preclinical and early translational studies provide meaningful biological clues worth noting. Phytochemicals such as curcumin, quercetin, and resveratrol have been shown to modulate PD-L1 expression on tumor cells, potentially enhancing T-cell activity and shifting the tumor microenvironment toward a more immune-permissive state. Similarly, emerging data highlight the gut microbiome as a critical mediator of immunotherapy response. Certain plant-derived compounds have been reported to enrich beneficial taxa such as *Akkermansia muciniphila*, which has been linked to improved outcomes with immune checkpoint inhibitors [40,41].

At present, these observations are based almost entirely on preclinical studies, and there is insufficient clinical evidence to support changes in patient management. These findings should therefore be interpreted cautiously and viewed as hypothesis-generating.

## 8. Clinical Management and Recommendations

Translating this complex and often incomplete evidence into practical clinical guidance is a major challenge. The key is not to memorize every possible interaction, but to adopt a structured approach built on communication, risk assessment, and shared decision-making.

### 8.1. Proactive Communication

The cornerstone of safe practice is proactive, non-judgmental communication. Since most patients will not volunteer this information, clinicians must take the lead and ask for the use of supplements at every visit. This means building standardized questions about all supplement use such as herbs, vitamins, and minerals into routine patient visits. The conversation must be framed with empathy, acknowledging the patient’s desire to be an active participant in their care. This trust is the foundation for the honest disclosure needed for any meaningful risk assessment.

### 8.2. Clinical Practice Guidelines and Reliable Resources

Oncologists are not expected to be herbalists, but they should know where to turn for reliable information. Both the Society for Integrative Oncology (SIO) and the American Society of Clinical Oncology (ASCO) have published clinical practice guidelines on this topic. These guidelines provide a framework for recommending safe complementary therapies while discouraging those with known risks. Point-of-care databases, like Memorial Sloan Kettering Cancer Center’s “About Herbs,” offer quick, evidence-based summaries to help assess risk in real time

### 8.3. Risk-Stratification Approach

A useful strategy is to stratify NPs based on their potential for harm, allowing for more nuanced guidance than a simple “yes” or “no”. The table below (Table 3) offers a clinical risk-stratification framework for common supplements. By focusing counseling on the highest-risk products while allowing for shared decision-making on lower-risk ones, clinicians can protect patient safety while honoring their autonomy. This stratification framework integrates both pharmacokinetic (PK) and pharmacodynamic (PD) considerations to categorize supplements based on their predominant mechanism of interaction.

Interindividual differences in drug-metabolizing enzymes can magnify NP–drug interactions. For instance, carriers of CYP3A5 or UGT1A1 28 alleles exhibit reduced clearance of many TKIs or irinotecan, respectively; concurrent intake of CYP3A4-inhibiting supplements may therefore precipitate toxicity. In such patients, ECG monitoring is advisable for agents with QT-prolongation risk (e.g., nilotinib + grapefruit). Personalized counseling based on genotype and comedication profile should be encouraged.

**Figure 2:**
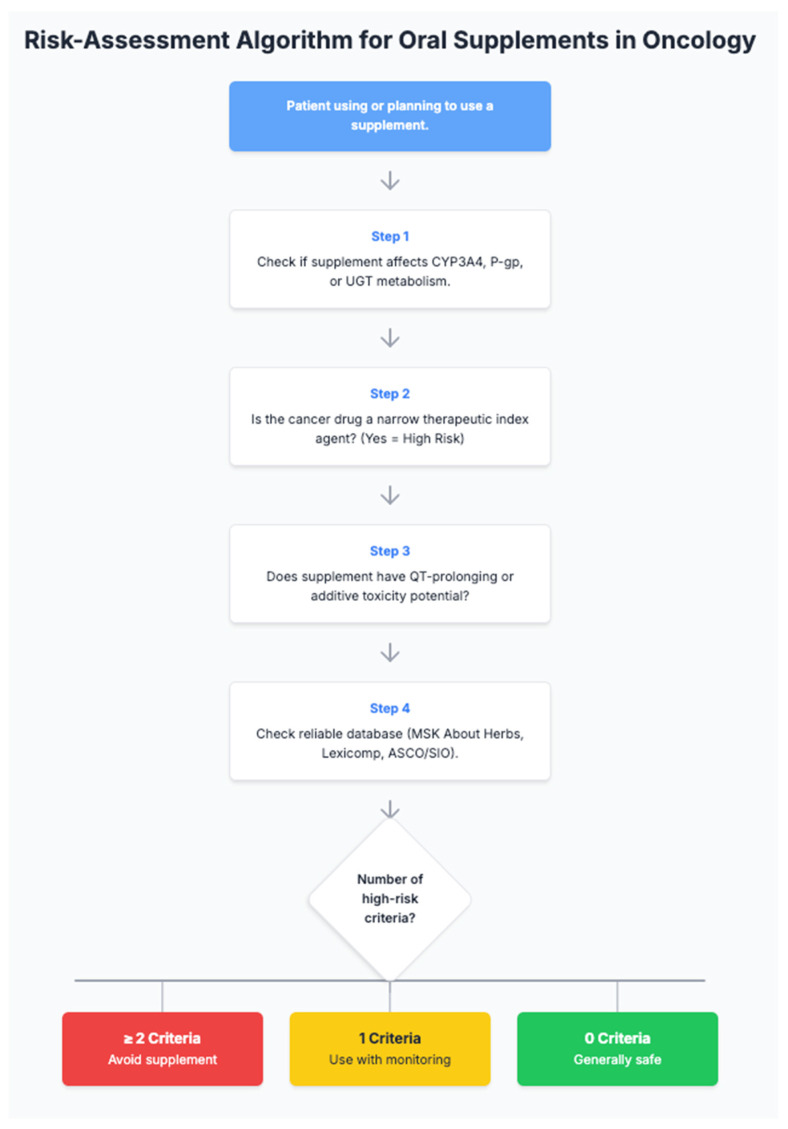
Simplified risk-assessment algorithm for oral supplement use in oncology.

A simplified clinical workflow for supplement risk evaluation is summarized in Figure 2.

### 8.4. Challenges in Pharmacovigilance

One of the greatest barriers to progress is the lack of systematic safety monitoring for dietary supplements. Unlike prescription drugs, they are not subject to rigorous pre-market safety testing, and post-market surveillance relies on a voluntary reporting system that is plagued by underreporting. This perpetuates the evidence gap and makes it nearly impossible to detect anything but the most severe interactions [46].

## 9. Conclusions

The intersection of natural products and cancer therapy involves intertwined pharmacokinetic (PK) and pharmacodynamic (PD) processes that can amplify or mitigate drug effects. The evidence is clear: natural products (NP)s are not benign substances but biologically active compounds capable of causing profound interactions with anticancer drugs. While some, like St. John’s wort, should be universally avoided, the reality for most products is a landscape of uncertainty, filled with conflicting or preliminary data.

To our knowledge, no other area of oncology practice is as affected by this mix of widespread patient use and sparse clinical data. We need well-designed clinical trials, but it is unrealistic to study every product this way. We must therefore become smarter, using real-world data from electronic health records and patient registries to find safety signals that smaller trials miss. Future computational tools may also help us predict these interactions [24].

Ultimately, safeguarding patient safety requires a cultural shift in oncology care. Rather than treating NP use as a hidden variable, it must become an open and integral part of clinical discussion. Through proactive communication, evidence-based guidance, and shared decision-making, clinicians and patients can work together to optimize therapeutic outcomes. By embracing rigorous science and fostering transparency, the field can move from reactive risk management to proactive, evidence-informed care.

### Natural Products in Cancer Prevention: The Mediterranean and Atlantic Diets

Although this review primarily focuses on therapeutic interactions between natural products (NPs) and cancer therapies, it is also relevant to acknowledge their preventive role when consumed within balanced dietary patterns. The Mediterranean and Atlantic diets, rich in polyphenols, flavones, carotenoids, and terpenes, illustrate this concept. Emphasizing fruits, vegetables, olive oil, whole grains, nuts, and moderate fish intake, these diets help reduce oxidative stress, modulate inflammation, and support metabolic health [47,48].

Epidemiologic evidence links adherence to these diets with reduced incidence of cancers related to oxidative and metabolic stress, notably breast and colorectal cancer [49,50]. Unlike concentrated supplements, dietary intake delivers bioactive compounds at physiological levels, maintaining safety while enhancing cellular defense mechanisms. Recognizing this continuum between prevention and therapy reinforces the role of evidence-based dietary patterns in integrative oncology.

## 10. Knowledge Gaps and Future Perspectives

Future research should prioritize pharmacoepidemiological studies leveraging real-world data sources such as EHR-linked registries, standardized adverse event reporting systems, and predictive computational models. These strategies can help bridge the persistent gap between preclinical mechanistic insights and clinically meaningful evidence. Despite substantial advances in mechanistic understanding, translation to clinical practice remains limited, and few prospective studies have quantified the real-world incidence or outcomes of natural product–drug interactions in oncology. Key priorities include well-powered pharmacokinetic (PK) studies of high-risk supplements, registry-linked pharmacoepidemiology to detect rare interactions, randomized trials with predefined clinical endpoints, and integration of pharmacogenomic and microbiome data to refine risk stratification.

Given the immunomodulatory potential of many natural products, patients receiving immune checkpoint inhibitors should be explicitly counseled to avoid unsupervised self-supplementation. Ultimately, the strength of this review lies not only in synthesizing mechanistic and clinical evidence, but in translating that knowledge into actionable insights. By situating herb–drug interactions within the daily therapeutic realities of modern oncology, particularly for patients treated with TKIs or ICIs, it shifts the focus from merely describing what is known to enabling more confident, evidence-informed decision-making at the bedside.

## Figures and Tables

**Table 1:** Key Pharmacodynamic Interactions of Natural Products in Oncology.

Natural Product	Anticancer Drug(s)	Interaction Type	Mechanism	Observed Outcome
Green Tea (EGCG) [12]	Bortezomib	Antagonistic	Direct binding of EGCG to the boronic acid group of bortezomib, forming an inactive complex.	Neutralization of proteasome inhibitory function, preventing cancer cell death.
Antioxidants (e.g., High dose Vitamin C, Vitamin E) [13]	Radiation Therapy, Alkylating agents, Platinum agents	Antagonistic (Theoretical)	Neutralization of reactive oxygen species (ROS) required for cytotoxicity.	Potential reduction in the efficacy of ROS dependent therapies.
Ginseng (*Panax ginseng*) [10]	5-Fluorouracil, Cisplatin, Docetaxel	Synergistic	Inhibition of pro survival pathways (e.g., NF-κB), induction of apoptosis, chemosensitization.	Enhanced cytotoxicity against various cancer cell lines in preclinical models.
Curcumin (*Curuma longa*) [14]	5-Fluorouracil	Toxicity Mitigation	Protective effect on intestinal mucosa, reducing inflammation and apoptosis in normal cells.	Amelioration of 5-FU-induced gastrointestinal toxicity (e.g., diarrhea, mucositis).
*Hangeshashinto* (TJ-14) [3]	Irinotecan, Fluoropyrimidines	Toxicity Mitigation	Modulation of inflammatory pathways and gut microbiota.	Reduced incidence and severity of chemotherapy induced diarrhea and oral mucositis.

**Table 2:** Therapeutic Benefit vs. Interaction Risk of Selected Natural Products.

Natural Product	Primary Clinical Indication/Benefit	Type of Evidence	Reported or Potential Risks	Mechanism of Interaction (PK/PD)	Clinical Consideration
Ginger (*Zingiber officinale*)	Reduction in chemotherapy-induced nausea and vomiting (CINV)	Multiple RCTs, meta-analyses	Mild antiplatelet effect at high doses	PD: antiplatelet	Generally safe at dietary doses; caution with anticoagulants
Ginseng (*Panax ginseng*)	Improvement of cancer-related fatigue	Large Phase III RCTs	CYP inhibition, hepatotoxicity (rare), antiplatelet effects	PK + PD	Use with caution, especially with TKIs or anticoagulants
Astragalus (*Astragalus membranaceus*)	Improved quality of life, appetite, and fatigue in NSCLC	Meta-analyses of RCTs in China	Minimal direct PK data; potential CYP interactions	Mostly theoretical PK	May be considered for supportive care; monitor for interactions
Curcumin (*Curcuma longa*)	Symptom relief, possible tumor sensitization	Preclinical + small clinical studies	Antioxidant antagonism, CYP modulation	PK + PD	Not recommended with ROS-dependent chemotherapy
Medicinal mushrooms (e.g., PSK from *Trametes versicolor*)	Adjunct to improve immune response and survival in GI cancers (Japan)	Clinical trials, regulatory approval in Japan	Limited PK data	Immunomodulation (PD)	Widely used as adjuvant in some countries

Evidence categories: RCTs = randomized controlled trials; PK = pharmacokinetic; PD = pharmacodynamic. Data reflect therapeutic intent and do not imply absence of interaction risk.

**Table 3:** A Clinical Risk-Stratification Framework for Common Natural Products in Oncology.

Natural Product	Level of Concern	Key Interacting Drug Classes	Primary Mechanism(s) of Concern	Management Recommendation	Clinical Action	Evidence Level
St. John’s Wort (*Hypericum perforatum*)	High	Chemotherapy (Irinotecan, Docetaxel), Targeted Therapy (TKIs)	Potent induction of CYP3A4 and P-gp	Avoid completely during and for several weeks before/after systemic cancer therapy.	Avoid use- Strong evidence supports contraindication (ASCO/SIO, PK Trials)	Strong clinical evidence
Green Tea Extract (high-dose EGCG) [42]	High	Proteasome Inhibitors (Bortezomib)	PD: Direct binding and inactivation of the drug	Avoid completely with bortezomib and other boronic acid-based inhibitors.	Avoid use during Proteasome Inhibitors therapy.	Preclinical + limited mechanistic human data
Grapefruit Juice [36]	High	Targeted Therapy (many TKIs, Sirolimus)	PK: Potent inhibition of intestinal CYP3A4	Avoid completely with oral CYP3A4 substrate drugs.	Avoid use- Strong evidence supports contraindication (ASCO/SIO, PK Trials)	Strong clinical evidence
Garlic (supplements) [29]	Moderate	Anticoagulants, Antiplatelet agents, Chemotherapy causing thrombocytopenia	PD: Antiplatelet effects; PK: Moderate CYP modulation	Avoid supplements, especially before surgery or in patients with low platelet counts. Culinary use is likely safe.	Use with Caution	Limited clinical/observational evidence
Ginseng (*Panax ginseng*) [10]	Moderate	TKIs (Imatinib), Anticoagulants, Hypoglycemic agents	PK: Inhibition of various CYP enzymes; PD: Hypoglycemic and antiplatelet effects	Use with caution. Monitor liver function, blood glucose, and coagulation parameters.	Use with Caution	Mixed clinical and preclinical studies
Curcumin (supplements) [43,44]	Moderate	Chemotherapy (Cyclophosphamide, Doxorubicin), Anticoagulants, Tamoxifen	PK: Complex CYP modulation; PD: Antioxidant and antiplatelet effects	Use with caution. Potential for antagonism with some agents. Evidence is conflicting.	Use with Caution	Preclinical/Small studies
Echinacea (*Echinacea purpurea*) [23]	Moderate	Immunosuppressants, Chemotherapy (Etoposide)	PK: Inhibition of CYP3A4; PD: Immunostimulatory effects	Use with caution. Avoid with immunosuppressants. Potential to increase toxicity of etoposide.	Use with Caution	Limited Clinical/mostly PK Studies
Ginger (*Zingiber officinale*) [45]	Low	Anticoagulants (at high doses)	PD: Mild antiplatelet effects	Generally safe at culinary doses for nausea. Use high-dose supplements with caution in patients on anticoagulants.	Generally Safe at dietary doses	Small clinical trials, Weak PK data

Strong clinical evidence: Randomized or controlled human PK (Pharmacokinetic) studies. Limited clinical: Small trials, case reports, observational data. Preclinical: In vitro, animal or mechanistic studies only. Mixed: Both preclinical and small clinical signals, but not definitive. Risk classification in Table 3 was developed through structured review of published clinical data, case reports, and systematic reviews, supplemented by expert consensus within the author team when data were incomplete. Use with Caution recommendations are based on concentrated supplement doses typically encountered in over-the-counter preparations (e.g., curcumin > 1 g/day extract, ginseng ≥ 1–2 g/day standardized root extract, garlic > 1200 mg/day capsules, grapefruit juice ≥ 8 oz/day, green tea extract > 800 mg/day EGCG equivalent). These reflect interactions that are often controversial or dosage-dependent and therefore require further validation. Dietary or culinary exposures usually fall below pharmacologically relevant thresholds.

## Data Availability

No new data were created or analyzed in this study. Data sharing is not applicable to this article.
